# LeafMachine: Using machine learning to automate leaf trait extraction from digitized herbarium specimens

**DOI:** 10.1002/aps3.11367

**Published:** 2020-07-01

**Authors:** William N. Weaver, Julienne Ng, Robert G. Laport

**Affiliations:** ^1^ Department of Ecology and Evolutionary Biology University of Colorado Boulder Boulder Colorado 80309 USA; ^2^ Present address: Department of Ecology and Evolutionary Biology University of Michigan Ann Arbor Michigan 48109 USA; ^3^ Department of Biology Rhodes College Memphis Tennessee 38112 USA

**Keywords:** computer vision, herbarium digitization, LeafMachine, leaf morphology, machine learning

## Abstract

**Premise:**

Obtaining phenotypic data from herbarium specimens can provide important insights into plant evolution and ecology but requires significant manual effort and time. Here, we present LeafMachine, an application designed to autonomously measure leaves from digitized herbarium specimens or leaf images using an ensemble of machine learning algorithms.

**Methods and Results:**

We trained LeafMachine on 2685 randomly sampled specimens from 138 herbaria and evaluated its performance on specimens spanning 20 diverse families and varying widely in resolution, quality, and layout. LeafMachine successfully extracted at least one leaf measurement from 82.0% and 60.8% of high‐ and low‐resolution images, respectively. Of the unmeasured specimens, only 0.9% and 2.1% of high‐ and low‐resolution images, respectively, were visually judged to have measurable leaves.

**Conclusions:**

This flexible autonomous tool has the potential to vastly increase available trait information from herbarium specimens, and inform a multitude of evolutionary and ecological studies.

The millions of plant specimens stored in herbaria around the world serve as priceless historical records of global biodiversity (Funk, 2003). The careful study and use of these specimens have been crucial in answering an assortment of evolutionary and ecological questions (Pyke and Ehrlich, 2010; Lavoie, 2013), such as the resolution of taxonomic puzzles and phylogenetic relationships (Ames and Spooner, 2008; Ng and Smith, 2016; Ng et al., 2019), phenological and species distribution responses to climate change (reviewed in Willis et al., 2017; Jones and Daehler, 2018; Lang et al., 2019), and plant–insect interactions through time (Lees et al., 2011; Meineke and Davies, 2018). Despite the clear value of herbaria, many collections have been neglected in recent decades as funding and curatorial expertise have waned (Dalton, 2003; Prather et al., 2004). Although this has the potential to lead to a tragic underutilization of accumulated scientific knowledge, recent worldwide efforts to digitize herbaria may mitigate these consequences by making troves of information easily available to researchers that was previously only accessible by physical visits to scattered repositories.

With the surfeit of available data represented by digitized specimens, the advent and development of computer vision and machine learning approaches provide promising solutions to efficiently procure information from otherwise unfathomable data sets (Gehan and Kellogg, 2017). For example, a number of machine learning tools have been developed to automate the identification of species from herbarium specimens (e.g., Unger et al., 2016; Wilf et al., 2016; Carranza‐Rojas et al., 2017), and these tools could be used to tackle the chronic backlog of unprocessed, unidentified, and misidentified plant specimens in herbaria. However, beyond their applications for species identification, digitized specimens harbor a wealth of available, but underutilized phenotypic information. Phenotypic trait measurements can provide important insights into the ecology and evolution of a species, but collecting these data manually can be a time‐consuming task. Multiple software solutions have been developed to rapidly capture plant phenotype information from digital images with minimal user input (e.g., LeafAnalyser, Weight et al., 2008; LeafProcessor, Backhaus et al., 2010; LeafJ, Maloof et al., 2013; Easy Leaf Area, Easlon and Bloom, 2014), including several applications that leverage the processing power of ubiquitous mobile devices for real‐time identification and/or feature extraction (e.g., LeafSnap, www.leafsnap.com; LeafScan, www.leafscanapp.com; Petiole, www.petioleapp.com). While employing varying degrees of automation, these applications largely still require manual user input or the creation of specifically prepared images (i.e., isolated leaves on a white background with a known scale) for accurate feature processing. Thus, fully automated computational approaches that can take advantage of the large number of already‐digitized herbarium specimens to efficiently and accurately identify and measure particular plant characteristics (e.g., leaf size, floral features) represent extremely valuable scientific tools that can overcome current limitations on data set inclusivity and magnitude.

Here, we describe the development of LeafMachine, an open‐source machine learning software package designed to autonomously identify, analyze, and extract trait information from digitized herbarium specimens or leaf images using a rigorous training and ground‐truthing regimen. LeafMachine is currently focused on extracting leaf area and perimeter, as these traits have been shown to provide important insights into the ecology and evolutionary history of plant species (e.g., Cornwell et al., 2014; Edwards et al., 2016). LeafMachine is designed to be flexible, accommodating the vast diversity of shapes, colors, textures, and associated materials on herbarium specimens (e.g., different species, preservation quality, mounting materials, labels, text, annotations). LeafMachine also has the potential to be adapted to identify and measure other plant traits (e.g., flowers, fruit, evidence of herbivory) or even traits associated with other organisms (e.g., lizard scales, butterfly wings).

## METHODS AND RESULTS

### LeafMachine overview

**Figure 1:**
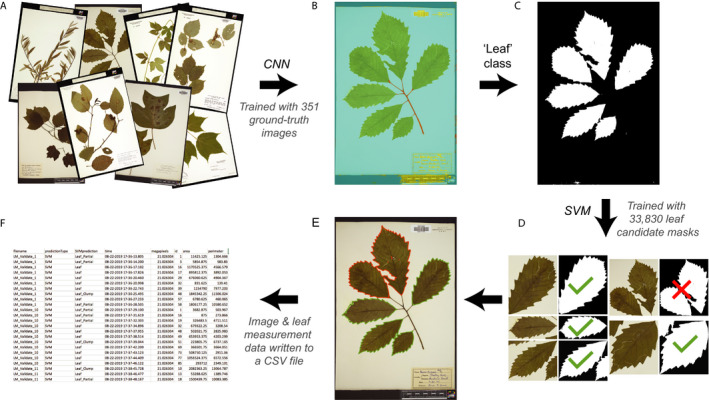
Workflow of LeafMachine to process a herbarium specimen image. (A) LeafMachine accepts images as a batch input. (B) With each image, LeafMachine uses a modified DeepLabV3+ convolutional neural network (CNN) to segment the image into five segmentation classes, including a ‘leaf’ class for identified leaves. The CNN was trained with 280 ground‐truth images and validated with 71 ground‐truth images. (C) The CNN outputs a binary mask for each segmentation class (‘leaf’ class shown). (D) With the ‘leaf’ class binary mask, LeafMachine then crops the image into different leaf candidate masks (LCMs), each only containing one binary component, and uses an AdaBoost support vector machine (SVM) to further classify LCMs as a single, measurable leaf (check mark), partial leaf (not shown), leaf clump (cross), or not a leaf (rejected; not shown). The SVM was trained using 33,830 leaf candidate masks that were extracted from the CNN training set and manually sorted into ‘leaf,’ ‘partial leaf,’ ‘leaf clump,’ or ‘reject’ categories. (E) LeafMachine then overlays the LCMs onto the original image as output for user verification, as well as (F) a CSV file containing leaf area and perimeter measurements, and processing information.

LeafMachine is a suite of machine learning algorithms that (i) identifies leaves and other components of a digitized herbarium specimen or leaf image using a pixel‐wise semantic segmentation convolutional neural network (CNN), and (ii) verifies whether each identified leaf is a single, measurable leaf using a support vector machine (SVM) algorithm (Fig. 1). We specifically focused our development and evaluation of LeafMachine to accurately identify and measure single leaves from a diverse selection of mostly perennial broad‐leaved angiosperms to reduce the number of preservation or architectural challenges that are common with grasses, herbs, gymnosperms, and other non‐flowering plants (e.g., small, obscure, folded, developmentally modified, or otherwise not obvious leaves). LeafMachine was developed in MATLAB (MathWorks, Natick, Massachusetts, USA), and can be readily implemented by users on computer systems running MATLAB (version R2019b or later) in Windows, Mac OS X, and Linux (example system configurations can be found in Appendix S1 and the user manual [available on GitHub, see Data Availability]). All LeafMachine algorithms can be downloaded from GitHub (https://github.com/Gene‐Weaver/LeafMachine/tree/V.2.0/Networks).

### Training the CNN to identify different specimen components

We randomly sampled digitized herbarium images to create a training data set. Although LeafMachine was not specifically designed to measure the morphological properties of grasses, herbs, and non‐flowering plants, we included taxa with these life histories in the training data set to maximize correct leaf classifications and enhance the generalizability of our machine learning algorithm. Some images were photographs taken in‐person at the Rocky Mountain (RM) and Missouri Botanical Garden (MO) herbaria (herbarium codes follow Thiers, 2020), while the rest were obtained from 136 well‐curated, digitized herbaria with Darwin Core files that were accessed through online consortiums (e.g., SERNEC, http://sernecportal.org/; SEINET, http://swbiodiversity.org/seinet/) using a custom MATLAB script (https://github.com/Gene‐Weaver/LeafMachine/blob/V.2.0/SandboxFunctions/downloadSubsetDWCimagesFixed.m) (Appendix S2). Herbarium collections ranged from 131 (WFU) to 429,181 imaged specimens (NLU). We downloaded high‐ and low‐resolution images, as defined by each herbarium (high = 2304 × 3072 pixels to 6879 × 9893 pixels, median = 3800 × 5700 pixels; low = 397 × 600 pixels to 2448 × 3264 pixels, median = 1400 × 1600 pixels), for 10 randomly chosen specimens from each herbarium (20 total images per herbarium). Our training set therefore captured a range of leaf shapes, phenological stages, preservation and image quality, lighting conditions, colors, orientations, mounting and labeling conventions, and specimen age (Appendix S3). Image files that were corrupted prior to or during download were deleted and not replaced, resulting in some specimens only having either a high‐ or low‐resolution image. This resulted in a total of 2685 training images (comprising both high‐ and low‐resolution images) that included ~1000 species spanning ~165 families (Appendix S4).

Pixel‐wise semantic segmentation algorithms require ground‐truth masks to instruct the neural network on how to classify each pixel in the training image. To create these, we subset the 2685‐image training set and manually classified the pixels of 425 images into one of five segmentation classes (‘leaf,’ ‘stem,’ ‘fruit/flower,’ ‘text,’ ‘background’). These segmentation classes represented apparent, functional definitions for machine learning purposes rather than strictly anatomically or developmentally correct botanical terms (e.g., grass leaf blades, conifer needles, and leaf petioles were typically classified as ‘stems’ in appearance for training the CNN because our focus was on identifying broad leaves). Although the current version of LeafMachine is focused on identifying leaves, we included non‐leaf segmentation classes to aid future development of LeafMachine’s capabilities to extract usable data from these other classes. We manually classified pixels by painting a colored layer over training images for each segmentation class with the MATLAB image labeler tool to signify that the painted pixels corresponded to the respective segmentation class. This resulted in ground‐truth images that were identical in dimension to the training image and that only contained the manually painted pixel masks for each segmentation class. Of the 425 ground‐truth images, we reserved 280 to train the CNN, 71 to validate the CNN during training, and 74 to evaluate the accuracy of the trained CNN algorithm (Appendix S4). Given that our training images ranged from 0.2–68.0 megapixels and training a CNN requires that images are all uniformly sized, we dynamically cropped each training image and its associated ground‐truth image into 360 × 360 pixel chips using a custom script (https://github.com/Gene‐Weaver/LeafMachine/blob/V.2.0/SandboxFunctions/dynamicCrop.m). The dynamic cropping process first crops a chip from the top left corner of the full image, then proceeds to crop in 180‐pixel sliding windows moving left to right until reaching the edge of the image. The process is then repeated 180 pixels below the first row until the entire training image has been cropped into 50% overlapping chips. This method produced 122,949 training chips and 20,422 validation chips, which became the training and validation input for the semantic segmentation algorithm.

We used a modified DeepLabV3+ CNN architecture for our semantic segmentation algorithm. The DeepLabV3+ CNN can achieve well‐resolved, computationally efficient segmentation masks around complex objects with the use of atrous separable convolutions and atrous spatial pyramid pooling (Chen et al., 2018). We further modified the algorithm to adjust the learning rate of the tensors in the segmentation to be weighted by the pixel frequency of each segmentation class for each training image. We also took advantage of transfer learning and spliced the pre‐trained feature extraction layers from a MATLAB‐provided ResNet18 network (included in the Deep Learning Toolbox) to enhance classification performance and reduce training time. Additional information about training LeafMachine can be found in the user manual (available on GitHub, see Data Availability).

To evaluate the accuracy of image segmentation by LeafMachine’s CNN, we calculated the proportion of pixels LeafMachine correctly predicted from the remaining 74 ground‐truthed images that were not used to train and validate the CNN. The global accuracy of the CNN across the five segmentation classes was 91.8%, although this figure is skewed by a disproportionately large number of ‘background’ pixels. Weighting the global accuracy by the proportion of ground‐truth pixels in each class, the intersection over union (IoU; also called the Jaccard index) was 88.8% and the mean IoU for the ‘leaf’ segmentation class was 55.2% (Table 1A). Additional information about LeafMachine’s CNN evaluation, including confusion matrices, can be found in the LeafMachine user manual (available on GitHub, see Data Availability).

**Table 1:** Evaluation of each step LeafMachine takes to process a herbarium specimen image (see also Fig. 1). (A) The accuracy of LeafMachine’s convolutional neural network (CNN) to segment specimen images into five classes (including a ‘leaf’ class) was calculated as the intersection over union (the proportion of pixels correctly predicted, weighted by the proportion of ground‐truth pixels in each class) using a set of 74 ground‐truthed images. (B) The accuracy of LeafMachine’s support vector machine (SVM) algorithm to identify single, measurable leaves from specimens was evaluated using 1000 randomly sampled high‐ and low‐resolution images from 21,316 processed specimens from SWMT and COLO representing 20 families. (C) LeafMachine’s leaf measurement accuracy was evaluated by processing 12 custom‐created herbarium specimen images (Appendix S5) and comparing LeafMachine leaf measurements to manual measurements in ImageJ. Comparing the binary masks from LeafMachine‐ and ImageJ‐processed images, 38 and 42 high‐ and low‐resolution leaves, respectively, were identified to be comparable and were used to assess differences in leaf measurements.

Steps taken by LeafMachine to process a specimen image	Evaluation
**(A) CNN** Identifying different specimen components (see also Fig. 1B)	Intersection over union All segmentation classes: 88.8% Leaf segmentation class: 55.2%
**(B) SVM** Identifying single leaves (see also Fig. 1D)	True positive Identified at least one leaf: 82.0% high‐resolution, 60.8% low‐resolution Identified all measurable leaves on a specimen: 42.4% high‐resolution, 39.1% low‐resolution False negative Misidentified leaf: 0.9% high‐resolution, 2.1% low‐resolution
**(C) Leaf measurements** Measuring leaves compared to ImageJ (see also Fig. 1F, Appendix S6)	Average difference High‐resolution: 145.4 mm^2^ (257.0 mm^2^ SD) Low‐resolution: 149.3 mm^2^ (250.9 mm^2^ SD)

### Training the SVM to identify single leaves

To create a training data set for the SVM, we ran the trained CNN on all 2685 images in the training data set. For each specimen, the CNN outputs five binary masks that correspond to the five segmentation classes. For those binary masks classified as belonging to the ‘leaf’ segmentation class, we cropped and saved each solitary object to create isolated binary mask objects (herein called *leaf candidate masks* [LCMs]). LeafMachine’s CNN produced 197,191 LCMs that we manually sorted into one of four categories: ‘leaf’ (6033 LCMs), ‘partial leaf’ (6375 LCMs), ‘leaf clump’ (1422 LCMs), or ‘reject’ (i.e., not a leaf; 183,361 LCMs). To reduce SVM bias toward the ‘reject’ category, we only included 20,000 random LCMs from the reject category during training (33,830 LCMs total). We chose 11 variables to train the SVM: nine computationally derived shape variables of the LCMs (area, perimeter, bounding box ratio, major axis length, minor axis length, eccentricity, equivalent diameter, extent, and roundness), one categorical variable (family name, if available), and one inherited variable (the original image size in megapixels; Di Ruberto and Putzu, 2014; Zhang et al., 2016). We used the MATLAB Classification Learner app to design an AdaBoost Decision Tree architecture with 20% holdout validation, a learning rate of 0.1, 50 splits, and 100 learners to decide on the validity of each LCM.

### Adjusting pixel classifications

Inaccurately classified pixels within a LCM, such as sections of a vein misclassified as a stem, will appear as holes in the final leaf prediction. We therefore provided a flood‐fill operation in LeafMachine (using the MatLab imfill() command) to fill all holes (including true holes that are the result of herbivory and specimen damage) occurring within the leaf boundary of the ‘leaf,’ ‘partial leaf,’ and ‘leaf clump’ categories to produce more refined masks.

### Evaluation of LeafMachine

We evaluated LeafMachine’s ability to (i) correctly identify leaves, and (ii) accurately measure identified leaves. To test LeafMachine’s ability to correctly identify single leaves, we ran the software on herbarium specimen images from the Rhodes College (SWMT) and University of Colorado Boulder (COLO) herbaria. We focused on 20 angiosperm families that primarily comprise trees, shrubs, and lianas to represent a mix of growth forms and life histories with generally simple broad leaves (but also include some herbaceous taxa, and taxa with compound leaves): Aceraceae, Adoxaceae, Anacardiaceae, Betulaceae, Cannabaceae, Caprifoliaceae, Ericaceae, Fagaceae, Lauraceae, Magnoliaceae, Malvaceae, Myrtaceae, Oleaceae, Platanaceae, Rhamnaceae, Salicaceae, Sapindaceae, Solanaceae, Ulmaceae, and Vitaceae (Table 2). This resulted in 718 and 9970 processed specimens from SWMT and COLO, respectively. We ran LeafMachine on both high‐ and low‐resolution images of each specimen (SWMT: low = 1250 × 1875 pixels, high = 4000 × 6000 pixels; COLO: low = 1400 × 2100 pixels, high = 3744 × 5616 pixels), resulting in a total of 21,316 processed images. This required 631 compute hours across a variety of computational resources (image processing times for example computer configurations can be found in Appendix S1). LeafMachine made at least one leaf measurement for 78.9% of processed images. Only 1.5% of the high‐resolution and 3.9% of the low‐resolution images did not have any ‘leaf,’ ‘partial leaf,’ or ‘leaf clump’ categories identified, with most of these images being specimens without leaves (e.g., early phenological stages of bud break/leaf out), or photographs or seed packets pasted onto a specimen sheet. Of the families processed, Malvaceae and Salicaceae specimens had the highest proportion of measurable leaves at high resolutions, while Solanaceae and Sapindaceae had the lowest proportion of measurable leaves (Table 2).

**Table 2:** The 20 angiosperm families used to test LeafMachine, the total number of digitized specimens for each family, and the proportion of specimens from which LeafMachine identified and measured at least one single leaf.^a^

Family	No. of digitized specimens	% images with at least one leaf measurement	
		High‐resolution	Low‐resolution
Aceraceae	504	90.7	74.2
Adoxaceae	98	89.8	79.6
Anacardiaceae	456	93.0	75.9
Betulaceae	940	92.3	69.1
Cannabaceae	80	91.3	70.0
Caprifoliaceae	706	91.9	71.7
Ericaceae	508	88.4	69.3
Fagaceae	1507	81.8	61.6
Lauraceae	17	88.2	82.4
Magnoliaceae	25	80.0	100.0
Malvaceae	494	98.2	92.5
Myrtaceae	1	100.0	100.0
Oleaceae	488	96.9	84.4
Platanaceae	6	83.3	83.3
Rhamnaceae	724	96.8	83.1
Salicaceae	2534	98.4	80.7
Sapindaceae	84	72.6	91.7
Solanaceae	1148	79.0	33.1
Ulmaceae	29	86.2	96.6
Vitaceae	23	95.7	100.0

^a^ All digitized herbarium specimen images available for these families from the Rhodes College (SWMT) and the University of Colorado Boulder (COLO) herbaria were processed by LeafMachine.

**Figure 2:**
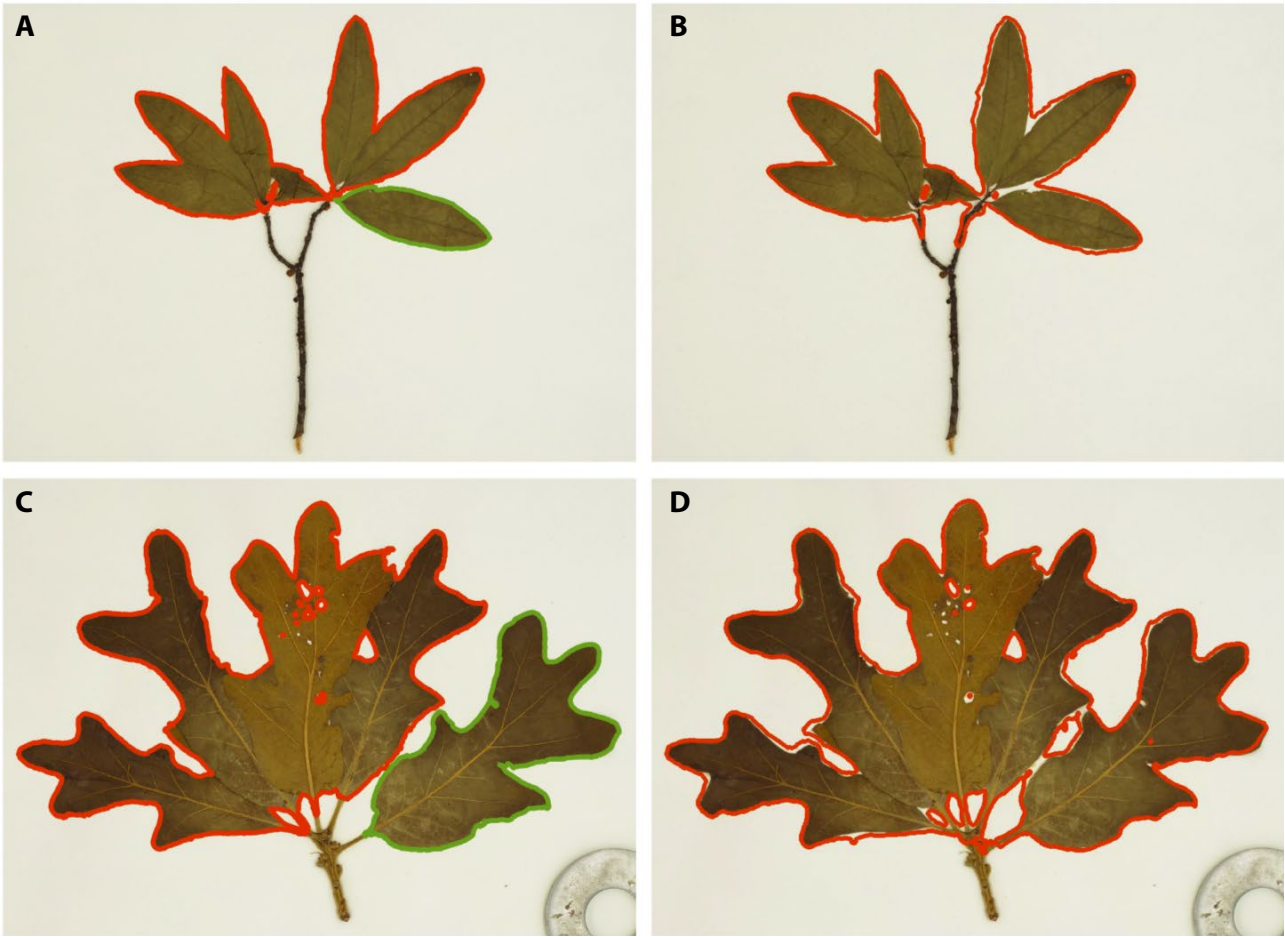
High‐ (A, C) and low‐ (B, D) resolution herbarium specimen images (Rhodes College herbarium barcode numbers SWMT09145 [A, B] and SWMT01894 [C, D]) processed by LeafMachine. In both high‐resolution images, LeafMachine identified that the specimens contained a single, measurable leaf (outlined in green; true positive) and a leaf clump (outlined in orange; true negative), while only a leaf clump was identified in the low‐resolution images (false negative). This reflects our general finding that higher‐resolution images result in better leaf identification and measurement by LeafMachine.

From the processed images, we evaluated LeafMachine’s ability to correctly identify leaves by randomly sampling 1000 high‐ and low‐resolution images in which at least one ‘leaf’ was identified, and 1000 high‐ and low‐resolution images in which a ‘partial leaf’ or ‘leaf clump’ was identified (4000 total images). The evaluated high‐ and low‐resolution images were paired to additionally evaluate LeafMachine’s performance on different image resolutions. From those images in which a ‘leaf’ was identified, we visually inspected each image to determine whether the ‘leaf’ identified by LeafMachine was indeed a correctly identified single leaf (true positive) or, instead, a ‘partial leaf,’ ‘leaf clump,’ or something else (false positive) (Fig. 2). We found that 82.0% of the high‐resolution and 60.8% of the low‐resolution images that contained measurable leaves (from visual assessment) had leaf morphometric information for at least one leaf (true positives) (Table 1B). For most images, LeafMachine identified and measured multiple leaves, with ~40% of all autonomous leaf identifications/measurements representing actual leaf measurements upon visual inspection (true positives; 42.4% high‐resolution, 39.1% low‐resolution; Table 1B). Many of the autonomous false positive leaf measurements appeared to result from issues with specimen preparation/preservation (e.g., leaf overlap, folding, degradation, breakage, or otherwise confusing leaf arrangements).

Similarly, we inspected each of the images in which a ‘partial leaf’ or ‘leaf clump’ was identified to determine whether a measurable leaf was actually present in the images (false negative) or not (true negative) (Fig. 2). We found that only 0.9% of high‐resolution and 2.1% of low‐resolution ‘partial leaf’ and ‘leaf clump’ images contained leaves visually assessed to be measurable (i.e., possessed at least one recognizable, intact, non‐overlapping leaf; false negative) (Table 1B). Otherwise, the majority of ‘partial leaf’ or ‘leaf clump’ images did not appear to be easily measurable by manual methods because of overlapping leaves or otherwise confusing leaf arrangements. Thus, LeafMachine was adequately capable of identifying and measuring leaves for the majority of herbarium specimens when such leaves were present.

To evaluate LeafMachine’s leaf measurement accuracy, we created 12 validation specimens by collecting, drying, and mounting leaves of several taxa with varying leaf sizes, shapes, textures, and colors on herbarium sheets with a ruler, label, and miscellaneous objects (e.g., washers, pens) often (or potentially) found in digitized herbarium images (Appendix S5). We photographed these validation specimens at COLO using standard digitization practices (Nelson et al., 2015) to obtain images at the median high and low resolutions of images in our training data set. We seeded these images into the set of SWMT and COLO images processed by LeafMachine and compared the leaf area measurements from LeafMachine to manual measurements obtained in ImageJ (version 1.52k; Schneider et al., 2012), software commonly used for leaf measurements. We measured leaves in ImageJ by converting images to 8‐bit color and used default thresholding to isolate leaves from the background. Individual leaf masks were selected with the wand tool and “filled” (Edit→Fill) prior to obtaining pixel areas via “Analyze Particles” (Reinking, 2007). We only compared measurements of those leaves in which LeafMachine created a binary mask that represented a whole, filled‐in leaf (i.e., the whole area within the leaf outline was measured). As LeafMachine currently only outputs measurements in pixels, we manually converted pixel measurements to square millimeters (mm^2^) using the ruler in each image.

Identification and measurement of validation image leaves proved more successful than the full herbarium image data sets, with successful measurements for all 12 images. For the high‐resolution images, 76.9% (40/52) of ‘leaves’ were correctly identified and measured, while 93.2% (41/44) of ‘leaves’ were correctly identified and measured in the low‐resolution images, indicating that simplified specimen images result in more reliable output than typical digitized herbarium specimen collections. The measurements produced by LeafMachine tended to be slightly smaller than those produced by ImageJ (38 comparable high‐resolution leaves: mean difference = 145.4 mm^2^, SD = 257.0 mm^2^; 42 comparable low‐resolution leaves: mean difference = 149.3 mm^2^, SD = 250.9 mm^2^; Table 1C, Appendix S6), likely due to differences in how leaves were thresholded in ImageJ and whether all pixels enclosed by the perimeter were included in the measurements or not. For example, measurements in ImageJ were influenced by shadows and fuzzy leaf boundaries, which differed between the high‐ and low‐resolution images. The same shadows and leaf boundaries appear to have been interpreted differently, and more conservatively, by LeafMachine. Additionally, we made considerable efforts in ImageJ to ensure all pixels enclosed by a leaf boundary were included in area measurements, sometimes manually filling initially unrecognized portions of leaves. LeafMachine made autonomous decisions about pixel inclusion, and may have excluded some pixels that differed qualitatively from included pixels, resulting in slightly lower estimates of leaf area even when the “fill holes” option was selected.

Despite the daunting challenges presented by variable image resolution among herbaria, broad variation among analyzed taxa, and the incredible diversity of specimen mounting/presentation techniques, LeafMachine was able to successfully identify and measure at least one leaf in the majority of analyzed images. The compounding of biological variation with specimen variability likely contributed to LeafMachine producing only half as many successful individual leaf measurements. Nevertheless, LeafMachine in its current form represents an amazingly flexible autonomous tool that can accommodate a range of broadleaf specimens typically found in herbarium collections for rapid, autonomous leaf measurement. This is especially true for high‐resolution images of taxa for which specimen images are more consistent, leaf margins are more clearly defined, leaf shape is more easily determined, and pixel characteristics can more confidently be classified as belonging to ‘leaf’ or ‘non‐leaf’ (e.g., Malvaceae, Salicaceae). We intentionally designed and trained LeafMachine to recognize the leaf morphometric features of primarily woody perennial angiosperm taxa with relatively simple leaf shapes. Yet, the inclusion of grasses, herbs, gymnosperms, and other non‐flowering plants in the training data set helped improve correct leaf classifications by presenting unusual shape masks (relative to our target taxa) to the algorithms that could be excluded as ‘non‐leaves,’ while at the same time providing the basis for the recognition of their morphological features that could broaden future applications of the software.

While we recognize the current limitations of LeafMachine, and strongly encourage manual verification of outputs, we also anticipate rapid refinement via improved training data and streamlined computational algorithms. Currently, LeafMachine struggles with images containing leaf overlap and folding, specimen breakage or other degradation, cluttered leaf arrangements, deeply lobed leaves, very large leaf blades, images with poor lighting or yellowed mounting paper, and extraneous materials (e.g., washers, fragment packets). We also documented differences in performance between high‐ and low‐resolution images, with higher resolutions generally resulting in better leaf identification (although not for our custom‐created validation specimens). Autonomous ruler identification and scale conversion also remain a challenge given the diversity, orientation, and placement of rulers in digitized herbarium specimens. The addition of an autonomous ruler detection feature, which is currently in development, will greatly streamline workflows. Specially prepared images, or data set customization for simpler herbarium images, could circumvent many of these issues, as well as ameliorate the biological and human‐introduced complexity contained within herbarium collections, resulting in more tractable subjects for the current version of LeafMachine. For example, we found that our algorithms performed exceptionally well in identifying and measuring leaf blades presented in uncomplicated settings, such as those represented in our 12 validation specimens. Carefully curated leaf specimen image sets would result in greatly improved identification and measurement performance of the software for individual users. Moreover, LeafMachine accurately identified the leaf blades of some taxa that were not included in the 20‐family test set (e.g., ferns; data not shown), suggesting that LeafMachine’s algorithms could be relatively easily optimized for identifying and measuring features of novel data sets with appropriate training data sets.

## CONCLUSIONS

Leaf area and shape have repeatedly been shown to provide insights into the ecology and evolutionary history of plant species (e.g., Cornwell et al., 2014; Edwards et al., 2016). Yet, obtaining leaf measurements that encapsulate individual‐, population‐, and species‐level differences is deceptively time‐consuming and labor‐intensive with currently available image processing software (e.g., ImageJ; tpsDig2, Rohlf, 2015; MASS, Chuanromanee et al., 2019), which rely heavily upon manual user input to specify leaf boundaries for measurement, or purpose‐built phenotyping software (Weight et al., 2008; Backhaus et al., 2010; Maloof et al., 2013; Easlon and Bloom, 2014) that require specially curated images for efficient processing. LeafMachine alleviates these tasks by leveraging machine learning to completely automate the process of extracting data from digitized herbarium specimens and leaf images for applications beyond species identification. Using a rigorous training regimen, individual leaf selection and measurement is automated via a CNN and SVM, with uncertainties and failures categorized for manual verification and processing. This allows users to quickly generate “first‐pass” data from large, historical data sets, while simultaneously allowing subsequent optimization for data generation on problematic specimen images. However, customized leaf image data sets could easily be designed to result in simpler image segmentation (e.g., similar to our validation specimens; Appendix S5), thus circumventing the major challenges facing current machine learning approaches, and allowing confident, rapid, and fully autonomous trait data acquisition.

By processing broad‐leaved angiosperm specimens across 20 families from two well‐curated digitized herbaria, which represent a diversity of life histories, growth forms, colors, textures, resolution, orientation, and mounting and labeling conventions, we demonstrated that LeafMachine can autonomously extract leaf morphometric data from digitized herbarium specimens in a time‐efficient manner similar to, or faster than, standard manual and computer‐aided methods. Although biological and human‐introduced variation in herbarium data sets present daunting challenges to this version of LeafMachine, we anticipate rapid development and improvement of the current application, and machine learning applications in general, that will result in significant performance enhancements in the next few years. These advancements will result in even greater time savings for large data sets and significantly streamlined workflows. Moreover, our novel application of machine learning has the potential to incorporate additional tools, such as autonomous ruler detection for scale, optical character recognition, and natural language processing, vastly increasing available trait information and specimen metadata to inform evolutionary and ecological studies.

## AUTHOR CONTRIBUTIONS

W.N.W. developed LeafMachine and wrote the accompanying user manual; R.G.L. conceived the idea for the software; J.N. and R.G.L. tested and provided feedback and direction on LeafMachine; J.N. and R.G.L. drafted the manuscript with input from W.N.W.

## Supporting information


**APPENDIX S1.** Hardware and operating system (OS) configurations of the five computers used to test LeafMachine.

**APPENDIX S2.** The high‐ and low‐resolution herbarium specimen images used to train LeafMachine’s convolutional neural network (CNN).

**APPENDIX S3.** Specimen age distribution of the 1343‐specimen (2685 images) training image data set. The oldest specimen was collected in 1845 and the most recent specimen was collected in 2017. Collection years were obtained from the Darwin Core files associated with each image; 579 specimens lacked collection dates.

**APPENDIX S4.** Counts of families, genera, and species included in the ground‐truth image data sets.

**APPENDIX S5.** Twelve custom‐created validation specimen images used to evaluate LeafMachine’s accuracy in leaf measurements (A–L).

**APPENDIX S6.** Differences in LeafMachine’s leaf area measurements compared to manual measurements in ImageJ for leaves from 12 custom‐created high‐ and low‐resolution herbarium specimen images (see also Appendix S5).

## Data Availability

LeafMachine was developed using MATLAB (version R2019b; MathWorks, Natick, Massachusetts, USA). The source code, software, and user manual are available at https://github.com/Gene‐Weaver/LeafMachine. The software can be implemented by end users in MATLAB.
