# Supplementary material for: LeafMachine: Using machine learning to automate leaf trait extraction from digitized herbarium specimens
Source: Appl Plant Sci. 2020 Jul 1;8(6):e11367. doi: 10.1002/aps3.11367 (PMC7328653; doi:10.1002/aps3.11367)
Supplement: Supplementary file 1 — APPENDIX S1. Hardware and operating system (OS) configurations of the five computers used to test LeafMachine. [file APS3-8-e11367-s001.docx]

**APPENDIX S1.** Hardware and operating system (OS) configurations of the five computers used to test LeafMachine.

| **Computer^a^** | **OS** | **CPU** | **System RAM** | **GPU (GPU-VRAM)** | **Storage** | **Avg. processing time (sec/specimen)^b^** | | |
| --- | --- | --- | --- | --- | --- | --- | --- | --- |
|  |  |  |  |  |  | **2 MP** | **20 MP** | **60 MP** |
| MacBook Air (2013) | macOS Mojave | Intel Core i5 (2.8 GHz) | 8 GB | Integrated Graphics (None) | 128 GB | 40 | 310 | 1149^c^ |
| MacBook Pro 15″(2017) | macOS Mojave | Intel Core i7 (3.1 GHz) | 16 GB | Integrated Graphics (None) | 1 TB | 14 | 114 | 250^c^ |
| High-end consumer PC | Windows 10 | Intel Core i7 8700k (4.8 GHz) | 32 GB | Nvidia GeForce RTX 2070  (8 GB) | 2 TB | 9 | 71 | 168^d^ |
| Mid-tier workstation | Windows 10 | Intel Xeon E3-1245 v6 (3.7 GHz) | 64 GB | Nvidia Quadro P4000  (8 GB) | 10 TB | 12 | 88 | 617^d^ |
| High-end workstation | Ubuntu 19.04 | Intel Xeon W-2145 (4.5 GHz) | 128 GB | Nvidia Quadro P6000  (24 GB) | 12 TB | 8 | 69 | 86 |

*Note:* GB = gigabyte; MP = megapixel; TB = terabyte.

^a^The MacBook computers lack a discrete GPU and could not take advantage of GPU acceleration.

^b^A standard processing time is listed for each configuration for three common megapixel ranges across the sampled herbaria.

^c^Exceeded system RAM and used swap memory to process images.

^d^Exceeded GPU-VRAM and used the CPU to process images.
