# Supplementary material for: LeafMachine: Using machine learning to automate leaf trait extraction from digitized herbarium specimens
Source: Appl Plant Sci. 2020 Jul 1;8(6):e11367. doi: 10.1002/aps3.11367 (PMC7328653; doi:10.1002/aps3.11367)

DOI

**APPENDIX S3.** Specimen age distribution of the 1343-specimen (2685 images) training image data set. The oldest specimen was collected in 1845 and the most recent specimen was collected in 2017. Collection years were obtained from the Darwin Core files associated with each image; 579 specimens lacked collection dates.

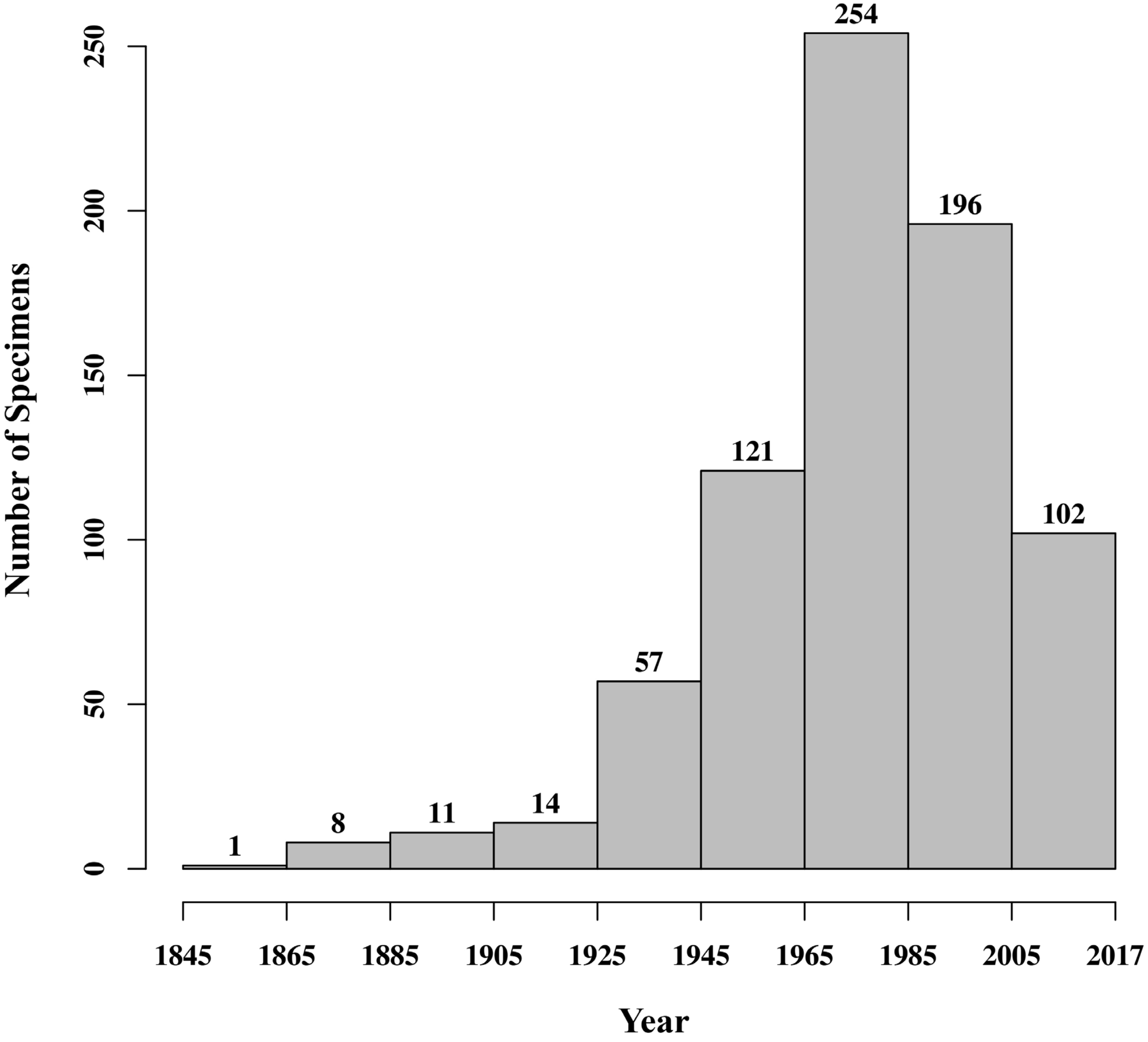

Supplement: Supplementary file 3 — APPENDIX S3. Specimen age distribution of the 1343‐specimen (2685 images) training image data set. The oldest specimen was collected in 1845 and the most recent specimen was collected in 2017. Collection years were obtained from the Darwin Core files associated with each image; 579 specimens lacked collection dates. [file APS3-8-e11367-s003.pdf]
