# Supplementary material for: LeafMachine: Using machine learning to automate leaf trait extraction from digitized herbarium specimens
Source: Appl Plant Sci. 2020 Jul 1;8(6):e11367. doi: 10.1002/aps3.11367 (PMC7328653; doi:10.1002/aps3.11367)
Supplement: Supplementary file 5 — APPENDIX S5. Twelve custom‐created validation specimen images used to evaluate LeafMachine’s accuracy in leaf measurements (A–L). [file APS3-8-e11367-s005.pdf]

~~DOI~~

APPENDIX S5. Twelve custom-created validation specimen images used to evaluate LeafMachine's accuracy in leaf measurements (A–L). These images include leaves from several broadleaf tree species representing a diversity of sizes, shapes, textures, and colors. An assortment of objects routinely found in digitized herbarium specimen images (e.g., washers, pens) are also included to validate LeafMachine's ability to ignore extraneous objects. These images are also available on GitHub (https://github.com/Gene-Weaver/LeafMachine/tree/V.2.0/Demo/Validation_Images).

A

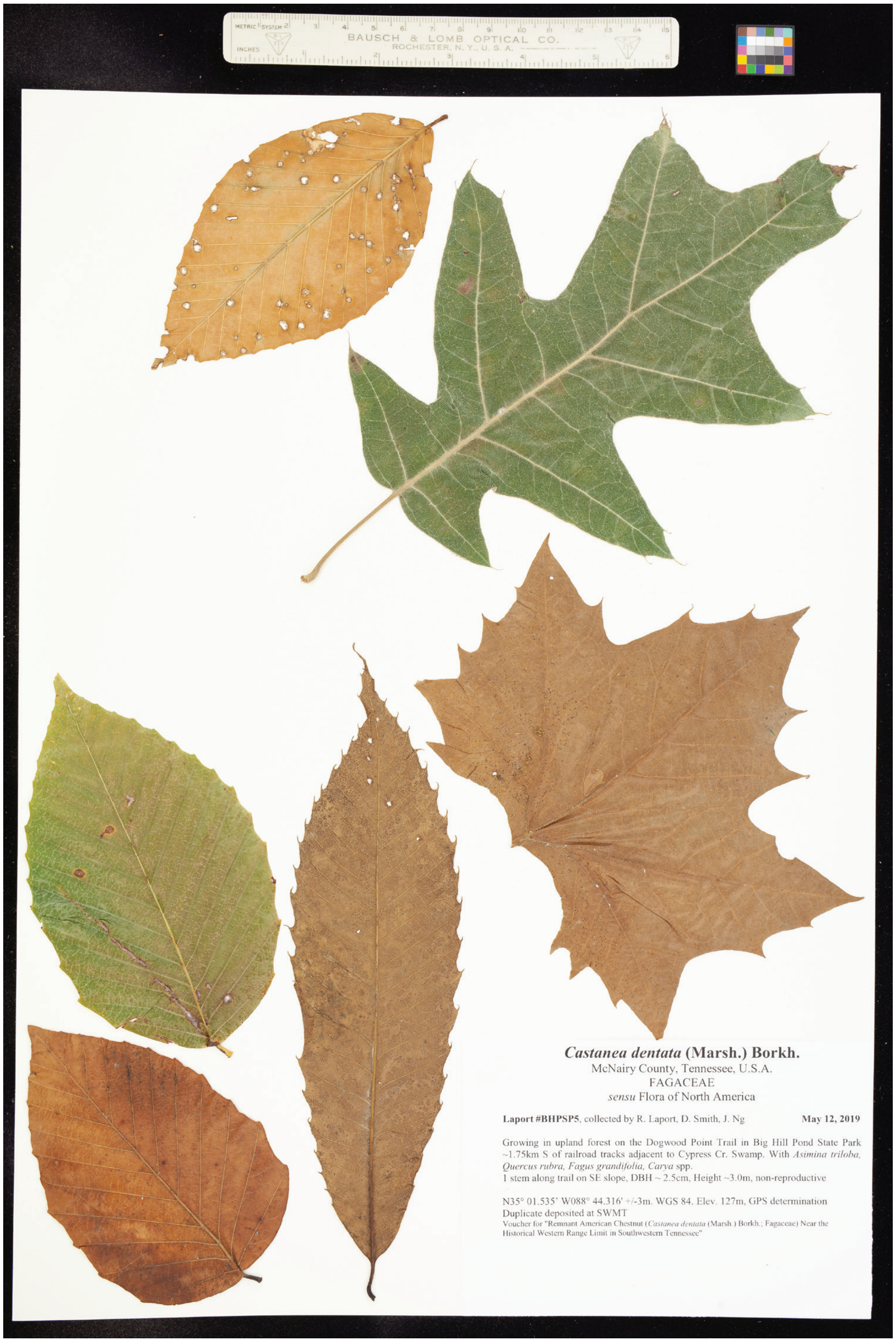

B

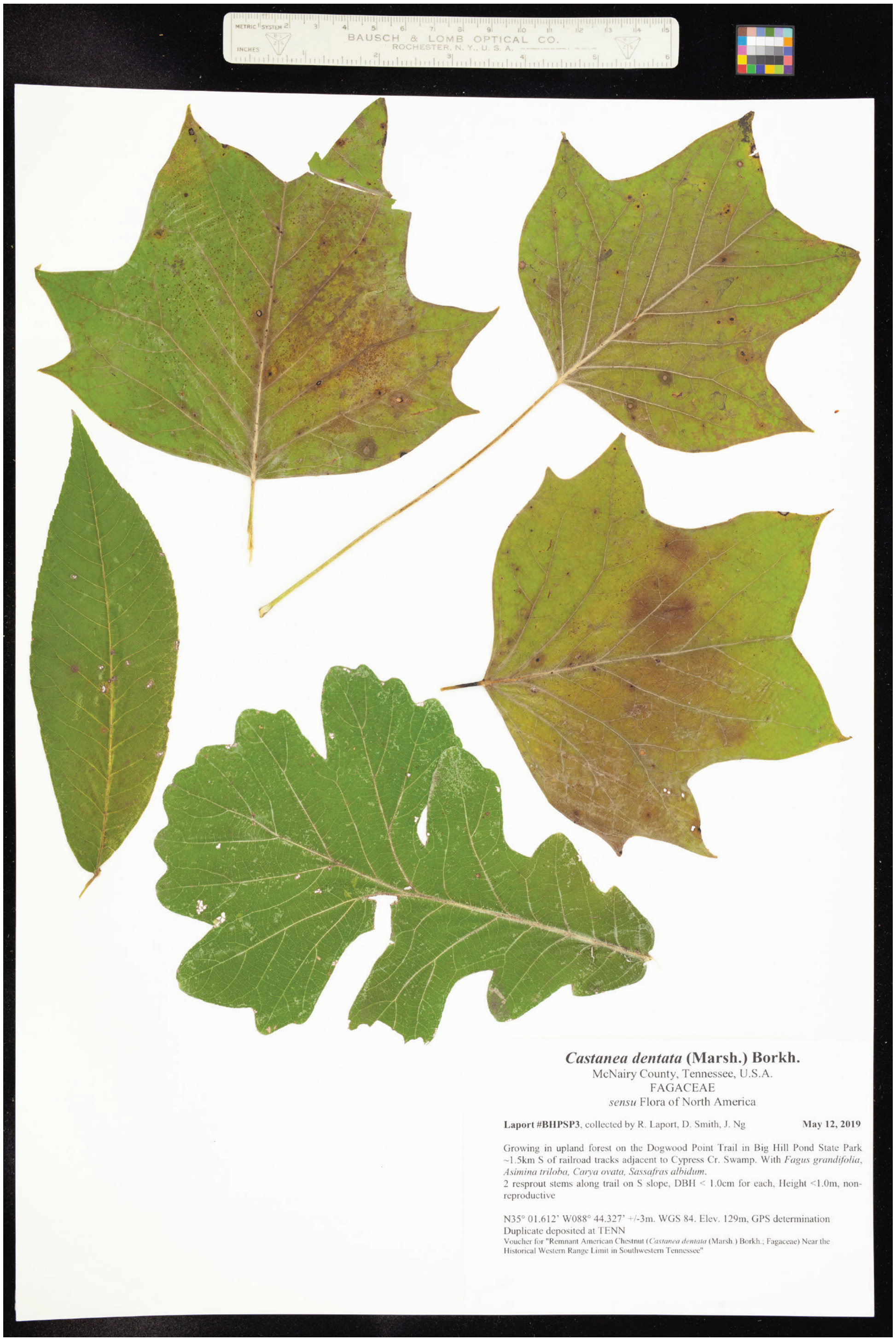
METRIC SYSTEM
BAUSCH & LOMB OPTICAL CO.
ROCHESTER, N. Y., U. S. A.
INCHES
Castanea dentata (Marsh.) Borkh.
McNairy County, Tennessee, U.S.A.
FAGACEAE
sensu Flora of North America
Laport #BHPSP3, collected by R. Laport, D. Smith, J. Ng **May 12, 2019**
Growing in upland forest on the Dogwood Point Trail in Big Hill Pond State Park ~1.5km S of railroad tracks adjacent to Cypress Cr. Swamp. With *Fagus grandifolia*, *Asimina triloba*, *Carya ovata*, *Sassafras albidum*.
2 resprout stems along trail on S slope, DBH < 1.0cm for each, Height <1.0m, non-reproductive
N35° 01.612’ W088° 44.327’ +/-3m. WGS 84. Elev. 129m, GPS determination
Duplicate deposited at TENN
Voucher for "Remnant American Chestnut (*Castanea dentata* (Marsh.) Borkh.; Fagaceae) Near the Historical Western Range Limit in Southwestern Tennessee"

C

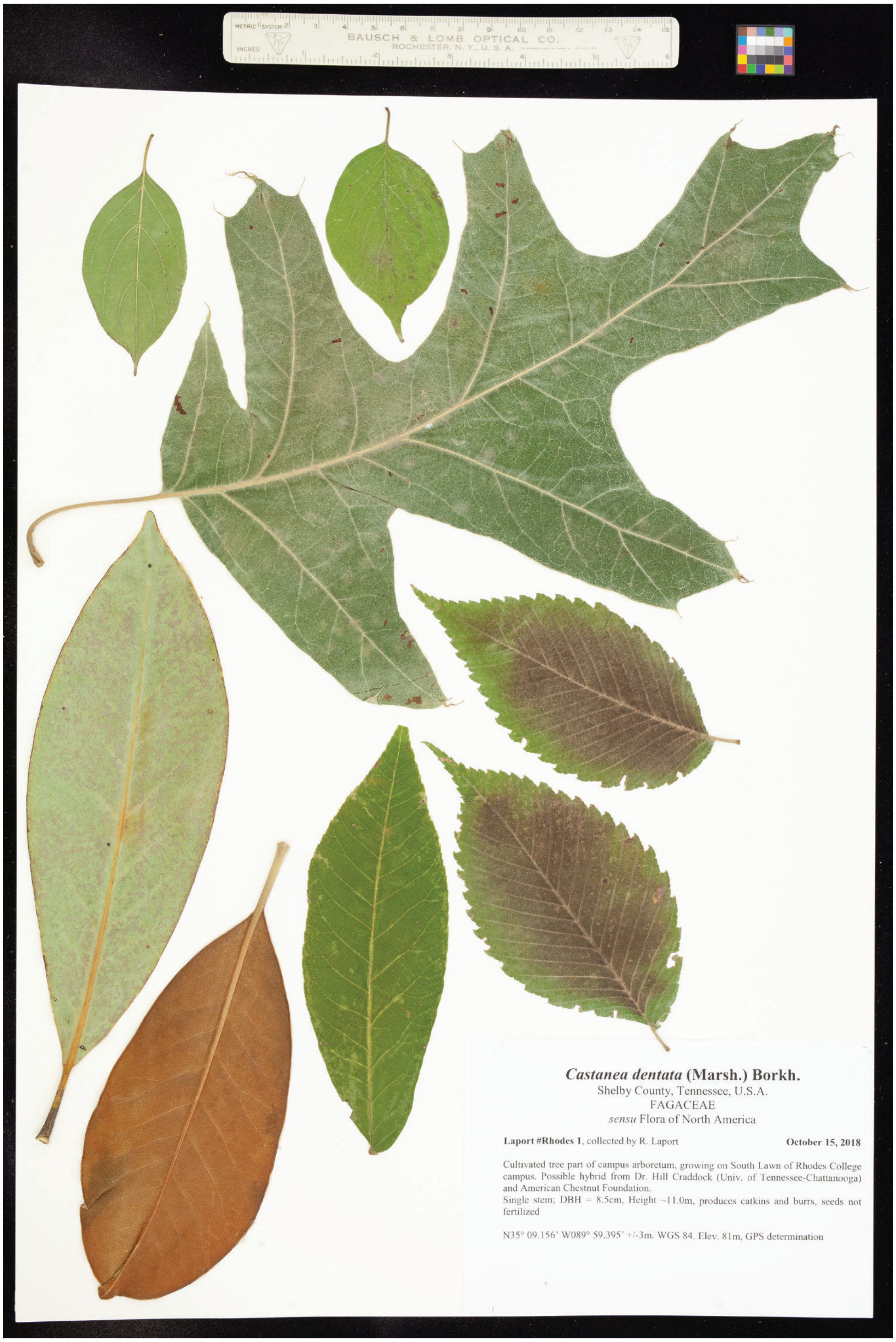
METRIC SYSTEM
BAUSCH & LOMB OPTICAL CO.
ROCHESTER, N. Y., U. S. A.
INCHES
Castanea dentata (Marsh.) Borkh.
Shelby County, Tennessee, U.S.A.
FAGACEAE
sensu Flora of North America
Laport #Rhodes 1, collected by R. Laport
October 15, 2018
Cultivated tree part of campus arboretum, growing on South Lawn of Rhodes College campus. Possible hybrid from Dr. Hill Craddock (Univ. of Tennessee-Chattanooga) and American Chestnut Foundation.
Single stem; DBH = 8.5cm, Height ~11.0m, produces catkins and burrs, seeds not fertilized
N35° 09.156' W089° 59.395' +/-3m. WGS 84. Elev. 81m, GPS determination

D

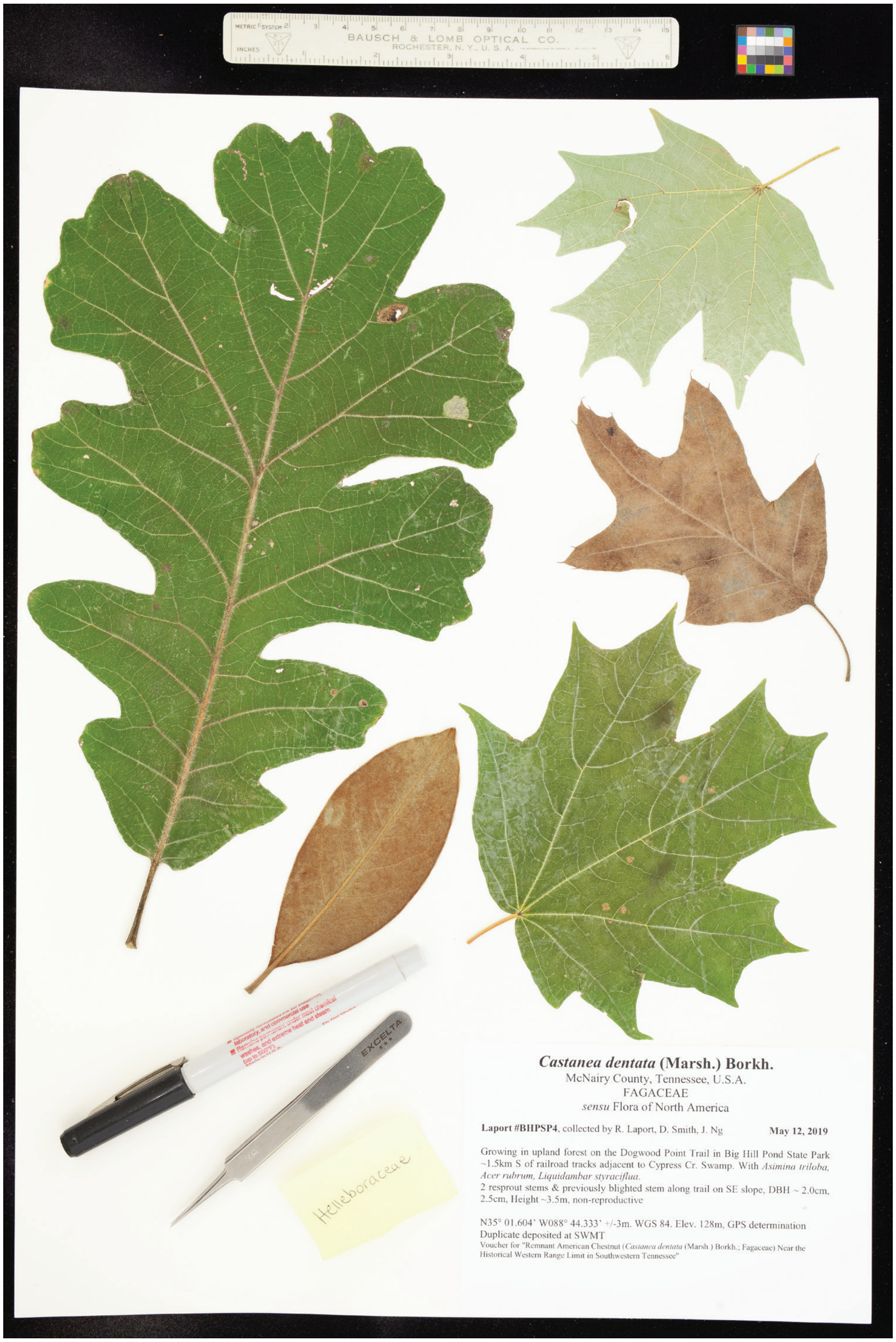
BAUSCH & LOMB OPTICAL CO.
ROCHESTER, N. Y., U. S. A.
Castanea dentata (Marsh.) Borkh.
McNairy County, Tennessee, U.S.A.
FAGACEAE
sensu Flora of North America
Laport #BHPSP4, collected by R. Laport, D. Smith, J. Ng
May 12, 2019
Growing in upland forest on the Dogwood Point Trail in Big Hill Pond State Park ~1.5km S of railroad tracks adjacent to Cypress Cr. Swamp. With Asimina triloba, Acer rubrum, Liquidambar styraciflua.
2 resprout stems & previously blighted stem along trail on SE slope, DBH ~ 2.0cm, 2.5cm, Height ~3.5m, non-reproductive
N35° 01.604' W088° 44.333' +/-3m. WGS 84. Elev. 128m, GPS determination
Duplicate deposited at SWMT
Voucher for "Remnant American Chestnut (Castanea dentata (Marsh.) Borkh.; Fagaceae) Near the Historical Western Range Limit in Southwestern Tennessee"
EXCELTA
Helleboraceae

E

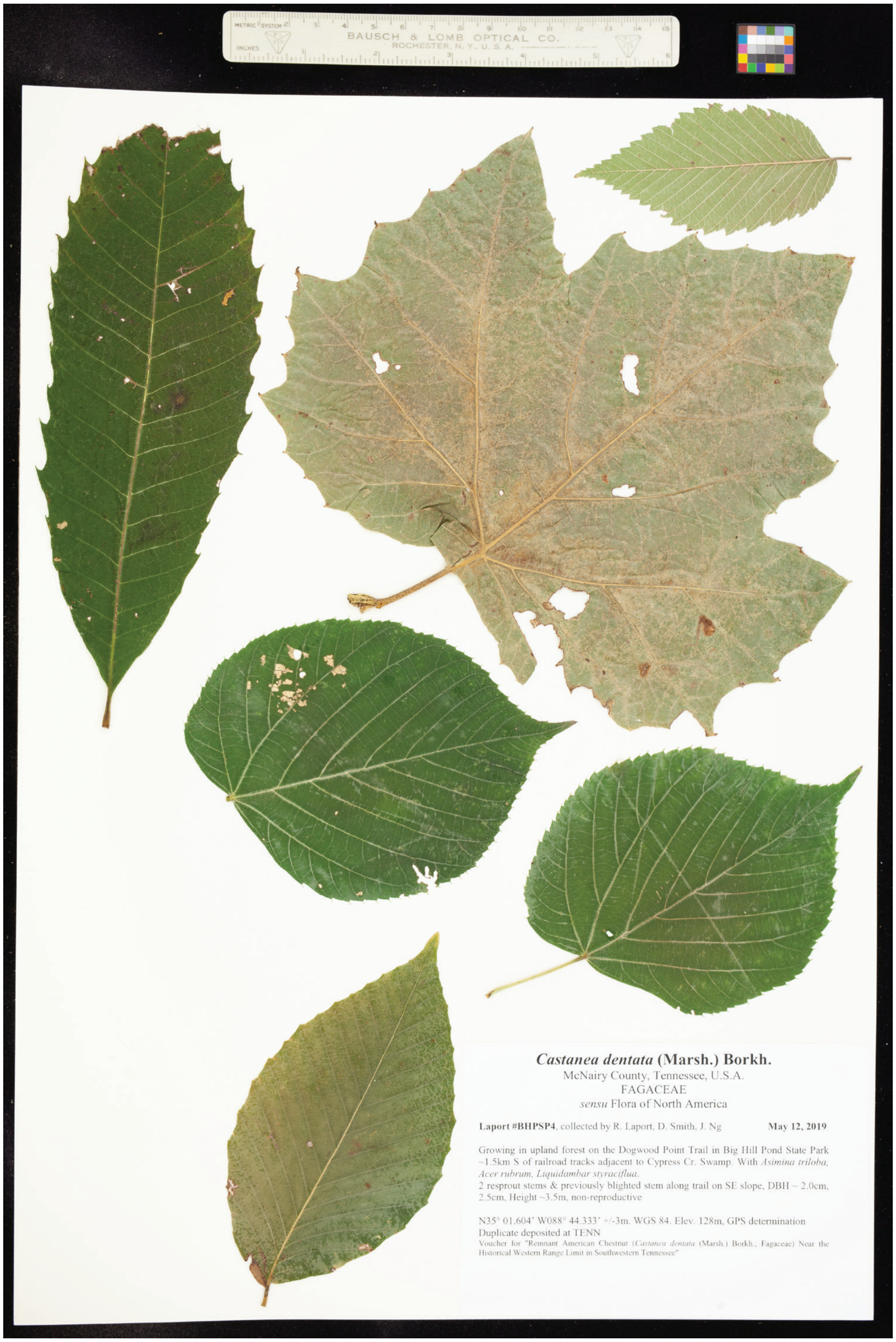
BAUSCH & LOMB OPTICAL CO.
ROCHESTER, N. Y., U. S. A.
Castanea dentata (Marsh.) Borkh.
McNairy County, Tennessee, U.S.A.
FAGACEAE
sensu Flora of North America
Laport #BHPSP4, collected by R. Laport, D. Smith, J. Ng
May 12, 2019
Growing in upland forest on the Dogwood Point Trail in Big Hill Pond State Park ~1.5km S of railroad tracks adjacent to Cypress Cr. Swamp. With *Asimina triloba, Acer rubrum, Liquidambar styraciflua.*
2 resprout stems & previously blighted stem along trail on SE slope, DBH ~ 2.0cm, 2.5cm, Height ~3.5m, non-reproductive
N35° 01.604' W088° 44.333' +/-3m. WGS 84. Elev. 128m, GPS determination
Duplicate deposited at TENN
Voucher for "Remnant American Chestnut (*Castanea dentata* (Marsh.) Borkh.; Fagaceae) Near the Historical Western Range Limit in Southwestern Tennessee"

F

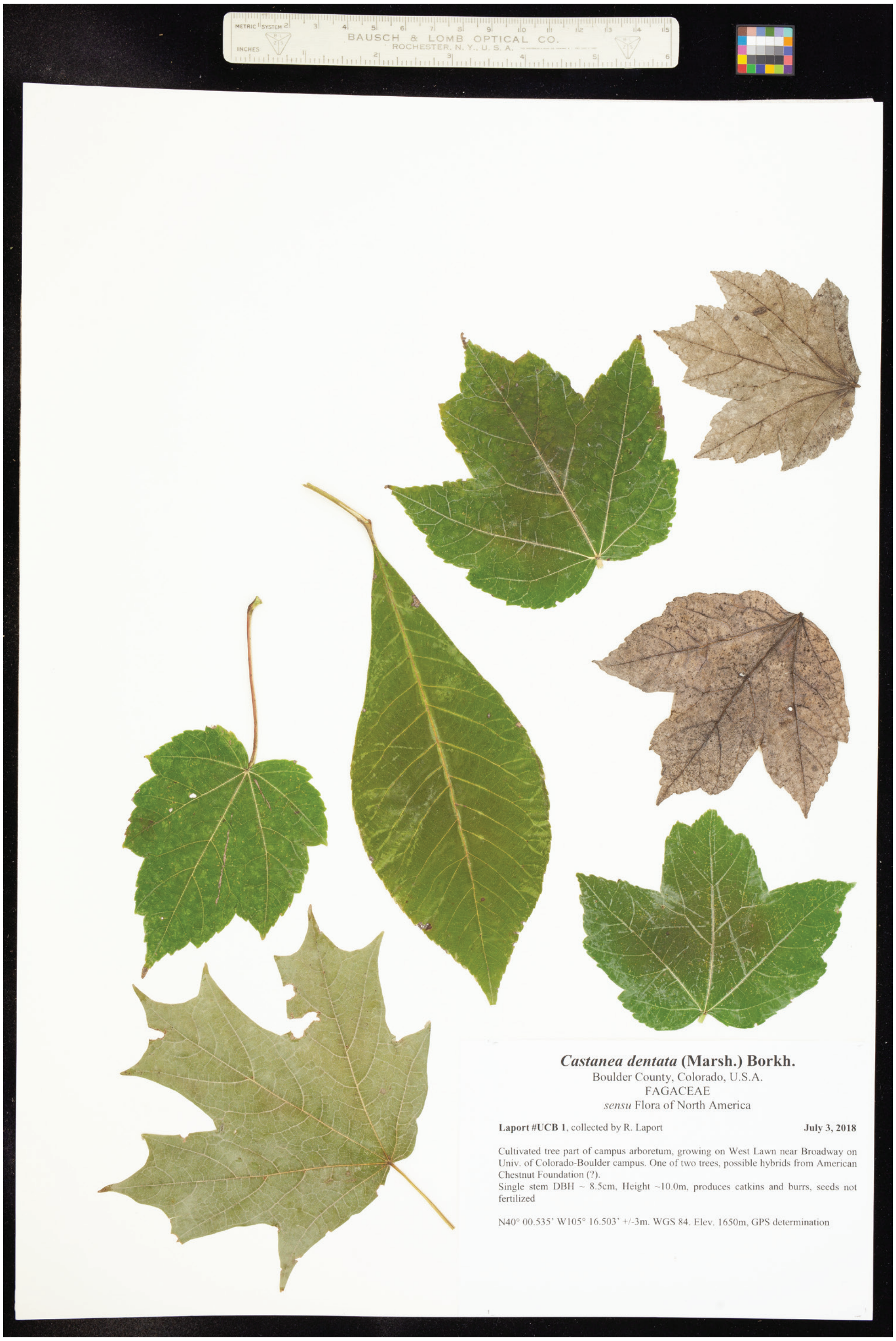
METRIC SYSTEM
INCHES
BAUSCH & LOMB OPTICAL CO.
ROCHESTER, N. Y., U. S. A.
Castanea dentata (Marsh.) Borkh.
Boulder County, Colorado, U.S.A.
FAGACEAE
sensu Flora of North America
Laport #UCB 1, collected by R. Laport
July 3, 2018
Cultivated tree part of campus arboretum, growing on West Lawn near Broadway on Univ. of Colorado-Boulder campus. One of two trees, possible hybrids from American Chestnut Foundation (?).
Single stem DBH ~ 8.5cm, Height ~10.0m, produces catkins and burrs, seeds not fertilized
N40° 00.535' W105° 16.503' +/-3m. WGS 84. Elev. 1650m, GPS determination

G

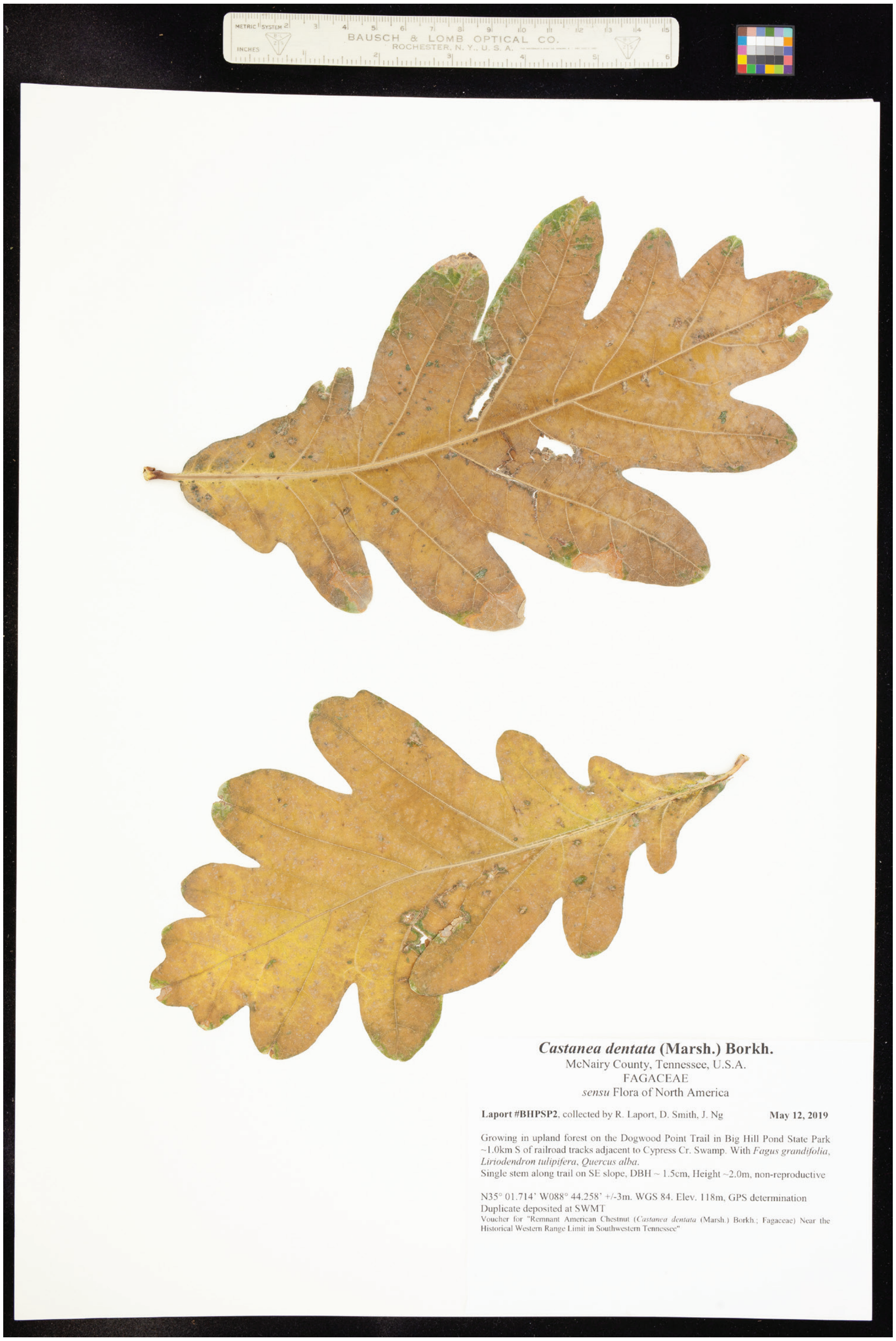
BAUSCH & LOMB OPTICAL CO.
ROCHESTER, N. Y., U. S. A.
Castanea dentata (Marsh.) Borkh.
McNairy County, Tennessee, U.S.A.
FAGACEAE
sensu Flora of North America
Laport #BHPSP2, collected by R. Laport, D. Smith, J. Ng
May 12, 2019
Growing in upland forest on the Dogwood Point Trail in Big Hill Pond State Park ~1.0km S of railroad tracks adjacent to Cypress Cr. Swamp. With Fagus grandifolia, Liriodendron tulipifera, Quercus alba.
Single stem along trail on SE slope, DBH ~ 1.5cm, Height ~2.0m, non-reproductive
N35° 01.714' W088° 44.258' +/-3m. WGS 84. Elev. 118m, GPS determination
Duplicate deposited at SWMT
Voucher for "Remnant American Chestnut (Castanea dentata (Marsh.) Borkh.; Fagaceae) Near the Historical Western Range Limit in Southwestern Tennessee"

H

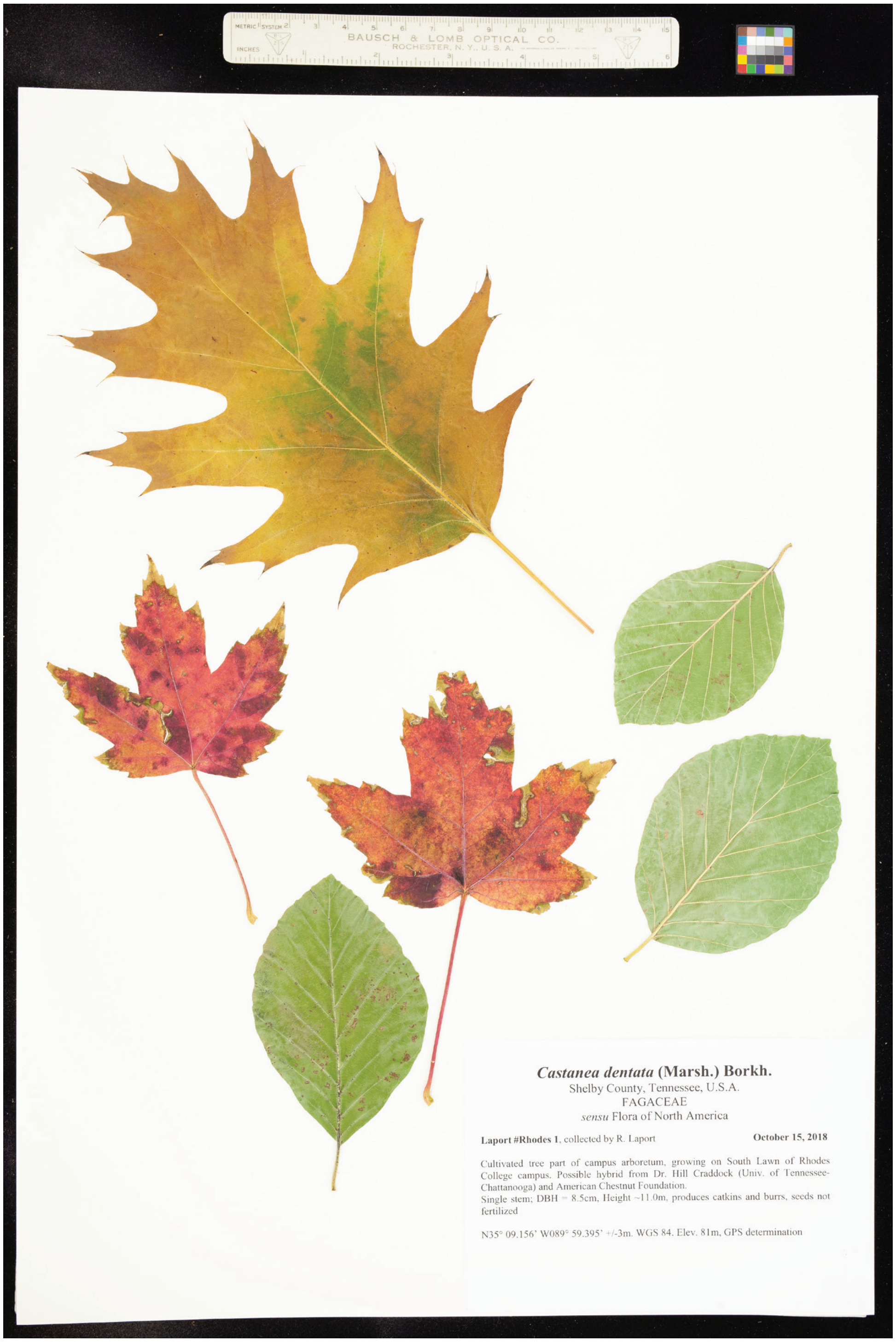
METRIC SYSTEM
BAUSCH & LOMB OPTICAL CO.
ROCHESTER, N. Y., U. S. A.
INCHES
Castanea dentata (Marsh.) Borkh.
Shelby County, Tennessee, U.S.A.
FAGACEAE
sensu Flora of North America
Laport #Rhodes 1, collected by R. Laport
October 15, 2018
Cultivated tree part of campus arboretum, growing on South Lawn of Rhodes College campus. Possible hybrid from Dr. Hill Craddock (Univ. of Tennessee-Chattanooga) and American Chestnut Foundation.
Single stem; DBH = 8.5cm, Height ~11.0m, produces catkins and burrs, seeds not fertilized
N35° 09.156’ W089° 59.395’ +/-3m. WGS 84. Elev. 81m, GPS determination

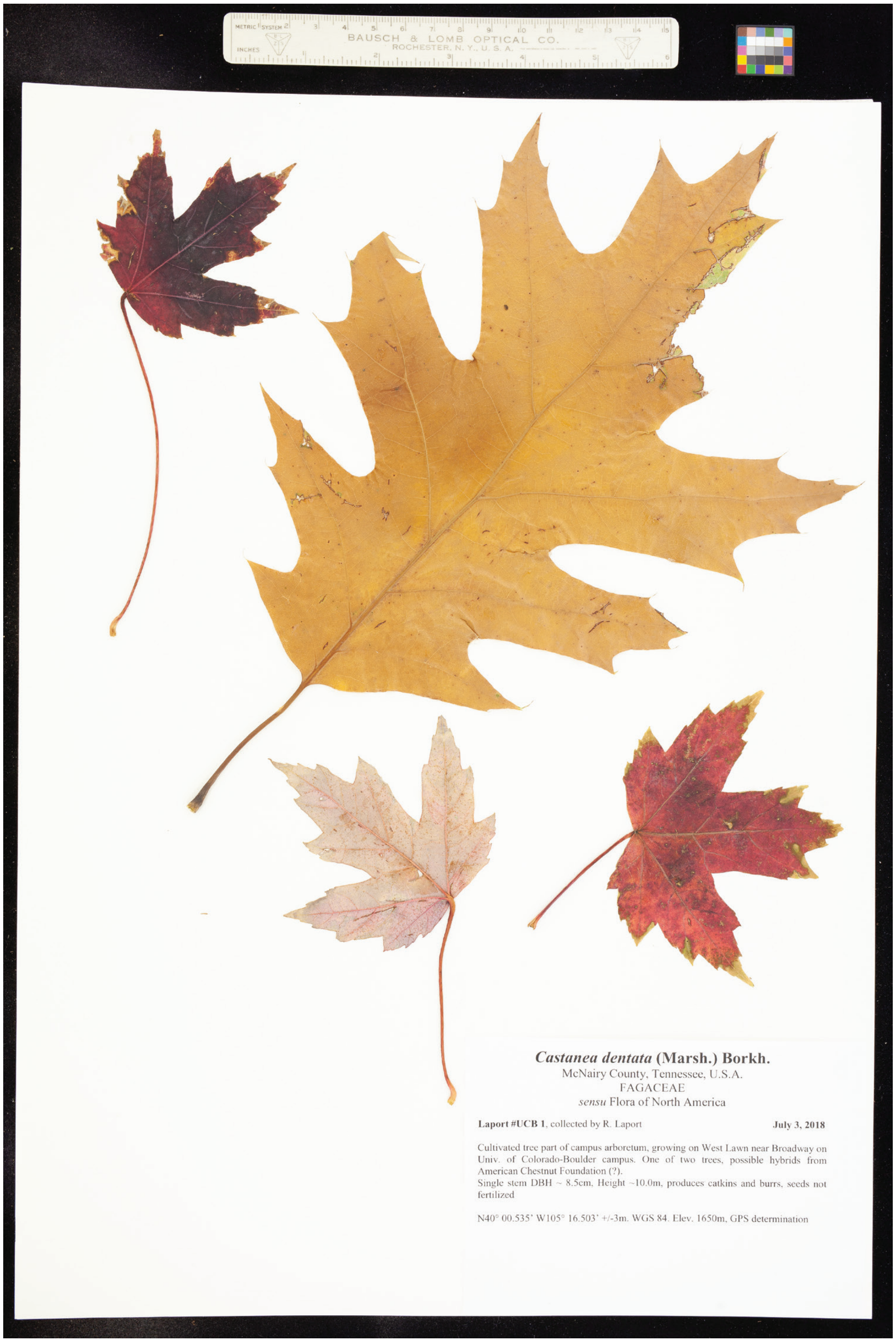
METRIC SYSTEM
BAUSCH & LOMB OPTICAL CO.
ROCHESTER, N.Y., U.S.A.
INCHES
Castanea dentata (Marsh.) Borkh.
McNairy County, Tennessee, U.S.A.
FAGACEAE
sensu Flora of North America
Laport #UCB 1, collected by R. Laport
July 3, 2018
Cultivated tree part of campus arboretum, growing on West Lawn near Broadway on Univ. of Colorado-Boulder campus. One of two trees, possible hybrids from American Chestnut Foundation (?).
Single stem DBH ~ 8.5cm, Height ~10.0m, produces catkins and burrs, seeds not fertilized
N40° 00.535' W105° 16.503' +/-3m. WGS 84. Elev. 1650m, GPS determination

J

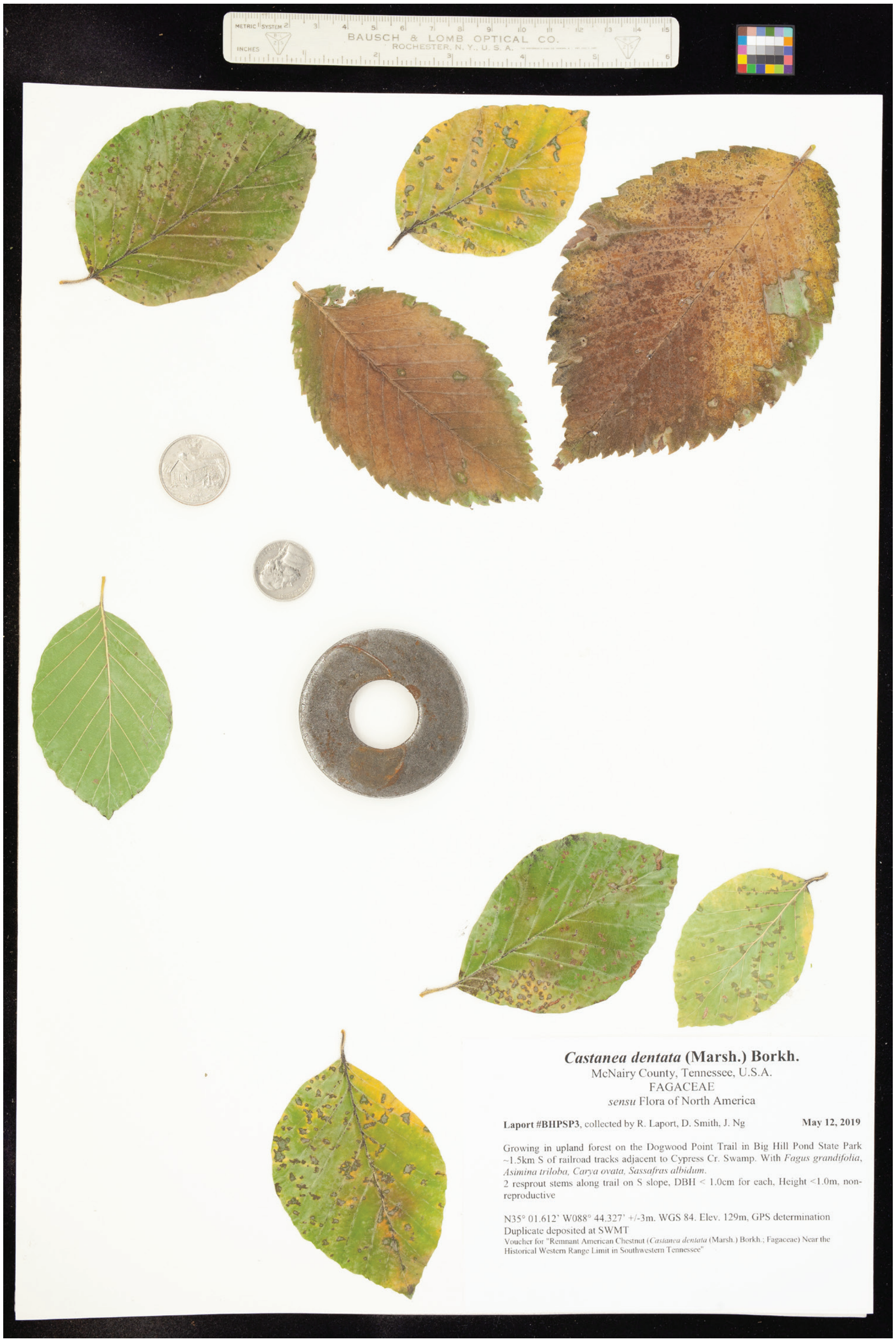
METRIC SYSTEM
BAUSCH & LOMB OPTICAL CO.
ROCHESTER, N. Y., U. S. A.
INCHES
Castanea dentata (Marsh.) Borkh.
McNairy County, Tennessee, U.S.A.
FAGACEAE
sensu Flora of North America
Laport #BHPSP3, collected by R. Laport, D. Smith, J. Ng
May 12, 2019
Growing in upland forest on the Dogwood Point Trail in Big Hill Pond State Park ~1.5km S of railroad tracks adjacent to Cypress Cr. Swamp. With *Fagus grandifolia*, *Asimina triloba*, *Carya ovata*, *Sassafras albidum*.
2 resprout stems along trail on S slope, DBH < 1.0cm for each, Height <1.0m, non-reproductive
N35° 01.612' W088° 44.327' +/-3m. WGS 84. Elev. 129m, GPS determination
Duplicate deposited at SWMT
Voucher for "Remnant American Chestnut (*Castanea dentata* (Marsh.) Borkh.; Fagaceae) Near the Historical Western Range Limit in Southwestern Tennessee"

K

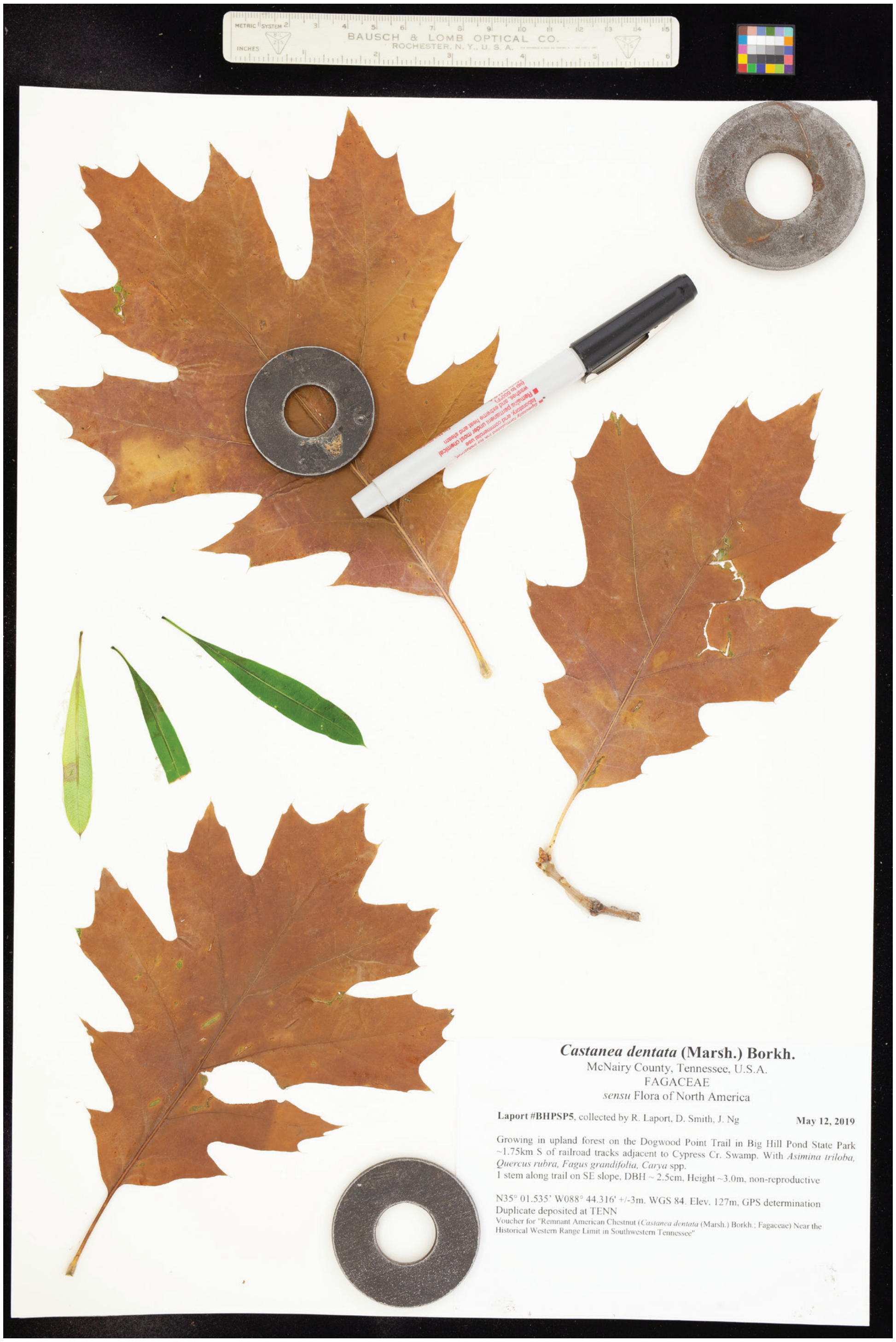
BAUSCH & LOMB OPTICAL CO.
ROCHESTER, N. Y., U. S. A.
METRIC SYSTEM
INCHES
Castanea dentata (Marsh.) Borkh.
McNairy County, Tennessee, U.S.A.
FAGACEAE
sensu Flora of North America
Laport #BHPSP5, collected by R. Laport, D. Smith, J. Ng
May 12, 2019
Growing in upland forest on the Dogwood Point Trail in Big Hill Pond State Park ~1.75km S of railroad tracks adjacent to Cypress Cr. Swamp. With *Asimina triloba, Quercus rubra, Fagus grandifolia, Carya* spp.
1 stem along trail on SE slope, DBH ~ 2.5cm, Height ~3.0m, non-reproductive
N35° 01.535' W088° 44.316' +/-3m. WGS 84. Elev. 127m, GPS determination
Duplicate deposited at TENN
Voucher for "Remnant American Chestnut (*Castanea dentata* (Marsh.) Borkh.; Fagaceae) Near the Historical Western Range Limit in Southwestern Tennessee"

DOI

L

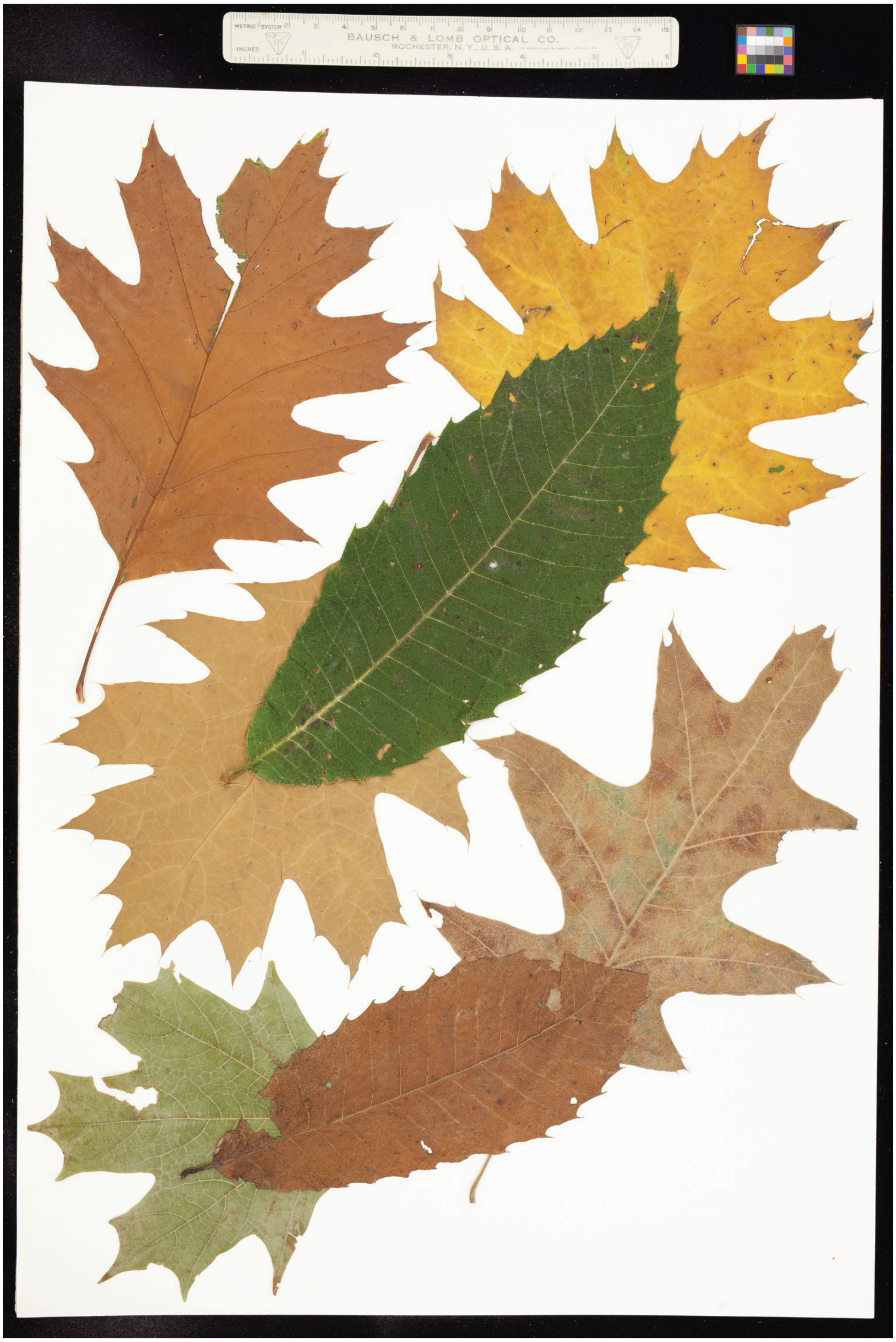
METRIC SYSTEM
BAUSCH & LOMB OPTICAL CO.
ROCHESTER, N. Y., U. S. A.
INCHES
