# Supplementary material for: LeafMachine: Using machine learning to automate leaf trait extraction from digitized herbarium specimens
Source: Appl Plant Sci. 2020 Jul 1;8(6):e11367. doi: 10.1002/aps3.11367 (PMC7328653; doi:10.1002/aps3.11367)

DOI

**APPENDIX S6.** Differences in LeafMachine's leaf area measurements compared to manual measurements in ImageJ for leaves from 12 custom-created high- and low-resolution herbarium specimen images (see also Appendix S5). Leaf measurement differences were calculated as (LeafMachine measurement – ImageJ measurement) for each leaf identified as having comparable binary masks (black circles).

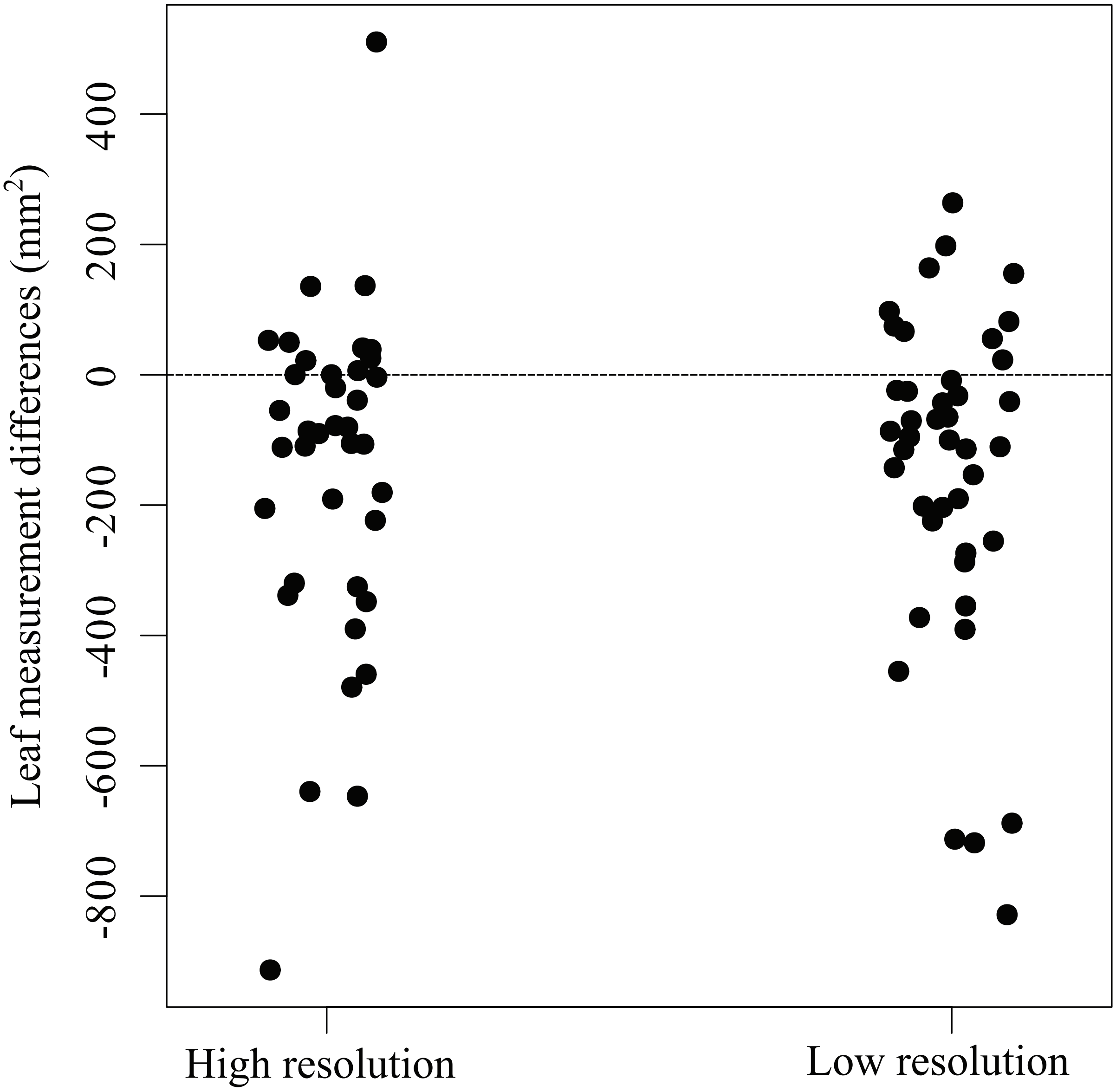

Supplement: Supplementary file 6 — APPENDIX S6. Differences in LeafMachine’s leaf area measurements compared to manual measurements in ImageJ for leaves from 12 custom‐created high‐ and low‐resolution herbarium specimen images (see also Appendix S5). [file APS3-8-e11367-s006.pdf]
